# Comparison of Lipid Properties and Cadmium and Lead Content in Red Deer (*Cervus elaphus*) Meat from Three Feeding Grounds

**DOI:** 10.3390/ani12202859

**Published:** 2022-10-20

**Authors:** Anna Kasprzyk, Janusz Kilar, Alina Walenia, Bożena Kusz

**Affiliations:** 1 Institute of Animal Breeding and Biodiversity Conservation, University of Life Sciences in Lublin, 13 Akademicka, 20-950 Lublin, Poland; 2 Institute of Agricultural and Forest Economy, Jan Grodek State University in Sanok, 21 Mickiewicza, 38-500 Sanok, Poland; 3 Institute of Economics and Finance, University of Rzeszow, 2 Ćwiklińskiej, 35-601 Rzeszow, Poland; 4 Department of Computer Science in Management, Faculty of Management, Rzeszow of Technology, 12 Al. Powstańców Warszawy, 35-959 Rzeszow, Poland

**Keywords:** *Cervus elaphus*, forest feeding grounds, organic farm and conventional farm feeding grounds, game meat, fatty acids, cholesterol, potentially toxic elements

## Abstract

**Simple Summary:**

As a product originating from the most natural living conditions with free foraging, red deer (*Cervus elaphus*) meat is becoming increasingly popular among consumers. A greater supply of this type of meat can be ensured by dynamically developing red deer farming on permanent grasslands. The study presents a comparison of the properties of lipids and the content of cadmium and lead in the LL and SM muscles of red deer kept in forest, organic farm, and conventional farm feeding grounds. The greater species and phytoncide diversity in the organic feeding ground enriched the quantitative profile and significantly increased the content of beneficial polyunsaturated fatty acids (FAs) (CLA—conjugated linoleic acid, ALA—alpha-linolenic acid, AA—arachidonic acid, and EPA—eicosapentaenoic acid) and the sum of polyunsaturated fatty acids (PUFAs), n-6 PUFA, and n-3 PUFA, in comparison with conventional farm and forest feeding grounds. It also contributed to a significantly lower n-6/n-3 ratio in both muscles. The study provides valuable data on the nutritional value and ecological safety of venison, which should be regarded as a health-enhancing food product. Furthermore, the present results can help breeders to make decisions about the locations of farms and the choice of red deer nutrition strategies.

**Abstract:**

The aim of the study was to compare the properties of lipids and the content of cadmium and lead in the meat of red deer (*Cervus elaphus*) kept in a forest feeding ground (FFG) and on organic and conventional farms in Poland. *Longissimus lumborum* (LL) and *Musculus semimembranosus* (SM) muscles were collected for the study from 36 red deer carcasses in an equal sex and age ratio (3 and 4 years old). From April to October, the animals consumed only the vegetation growing in the feeding grounds. The floristic and phytoncide composition, as well as the fodder value, of the feeding grounds were assessed. Additionally, the intramuscular fat and cholesterol content, the profile, sum, and ratios of fatty acids (FAs), lipid nutraceutical parameters, and cadmium and lead content were determined in LL and SM. The plant composition comprised 116 species in the organic feeding ground (OFG) and 78 species in the conventional feeding ground (CFG). The LL and SM muscles of the red deer from the organic and forest feeding grounds exhibited significantly lower amounts of fat and cholesterol than those from the conventional system. The differences in the fatty acid composition between the three groups were quite small. Differences in intramuscular fat content contributed to a higher percentage of PUFAs in the FFG and OFG, in comparison to the CFG. In all types of feeding ground, the cadmium (0.002 to 0.008 mg/kg) and lead (0.009 to 0.019 mg/kg) content determined in the meat did not pose a threat to consumers.

## 1. Introduction

In the twenty-first century, food no longer serves only human nutritional needs but has gained many new functions, defined by consumers. These include the support of health, maintenance of high physical and mental fitness as well as a healthy figure, extension of the lifespan, and pleasure associated with food consumption [1]. The shift towards the health-enhancing functions of food emphasizes the role of natural products, especially organic foods, whose availability and popularity are systematically increasing [2,3,4,5,6,7,8,9].

Meat has always been and still is an important part of the human diet, but the perception of its quality and choice-related attributes have been changing constantly [1,10,11,12]. Game meat from animals reared in the most natural production conditions and free foraging systems is part of an innovative meat consumption pattern [13,14,15,16,17,18], although the consumption of game meat harvested during hunting has been part of the human diet since ancient times. With its natural characteristic and chemical composition, game meat fully meets the biological needs of humans. It also has a unique aroma and flavor [14,19]. The determination and promotion of venison’s value may increase the consumption thereof, particularly in Poland, where it is very low [20]. In 2020, Polish consumers consumed only 0.084 kg of venison; the highest (0.273 kg/person) and the lowest (0.063 kg/person) consumption levels were recorded in 2016 and 2013, respectively [21]. Meat from free-living red deer is the most widely consumed product (46.12% in 2020) of all game meat produced in Poland [22]. In many countries, the demand for game meat exceeds the production capacity of free-living populations; hence, it has to be supplemented with farmed meat [20,23]. Farm rearing of red deer and fallow deer for meat originated in New Zealand [24]. In Poland, red deer farming is legally sanctioned and has been developing steadily since 2001 [25]. In 2010, there were 369 red deer and fallow deer farms in Poland. This number increased to 857 in 2021, with approximately 35,000 red deer reared on farms [26]. Many aspects have been addressed in investigations of the nutritional value and meat quality in free-living red deer [17,18,27,28,29,30], farmed deer [31,32,33,34], and both groups in comparative studies [23]. The effects of sex [17,18,23,28,34], age [27,28], diet [33], slaughter season [32], and country of origin [32], mainly on the fatty acid profile, were determined in these studies carried out on conventional farms. There is interest in the manipulation of the meat fatty acid profile, due to their possible beneficial and or detrimental action on human health. The present study was undertaken to fill the gap in the knowledge of the nutritional value of red deer meat from organic farms.

The aim of the study was to compare the properties of lipids and the content of cadmium and lead in the meat of red deer (*Cervus elaphus*) kept in forest feeding grounds and on organic and conventional farms. The nutritional contribution of venison to the requirements for fat, fatty acids, and cholesterol in the diet for adults was determined as well.

## 2. Materials and Methods

### 2.1. Animal Management

The animals came from three feeding grounds: forest (FFG), organic (OFG), and conventional (CFG). The feeding grounds provided the basic feed for all the animals. The forest feeding grounds and the red deer farms were located in Poland, in the north-eastern part of Podkarpackie Province. The forest feeding ground covered approximately 8000 ha and exhibited considerable species richness of trees, shrubs, shrublets, and meadow plants [35]. Agricultural crops also provided additional food for the red deer. Due to the size of the area and the feeding ground diversity, the composition and nutritional value of vegetation consumed by the free-living red deer was not controlled. The animals from the OFG were raised at a farm with an organic farming certificate [36,37]. The basic feed for the animals was provided by natural grazing ground. The density of red deer in the organic system was 0.20 LU (large livestock units)/ha. The animals were kept in their natural habitat, respecting the EU Directive 2010/63/EU. In winter, the OFG deer received certified feed from their farm, i.e., hay, straw, carrots, grain of oats, and corn, ad libitum. In the conventional system, the stock density of 0.52 LU/ha was applied, according to the DEFRA [38] and FEDFA [39] recommendations. In winter, they were additionally fed with haylage, straw, and fodder beets ad libitum. Constant accessibility to water and salt licks was provided on both farms.

### 2.2. Vegetation and Nutritional Value of Farm Feeding Grounds

The floristic analyses of the feeding grounds (OFG and CFG) were carried out once a month between April and October. In this period, their vegetation was the only fodder available to the animals. In each feeding ground, 35 phytosociological relevés were made using the Braun–Blanquet method [40]. Plant species were identified with the use of the guide compiled by Matuszkiewicz [41]. Based on the monthly analyses, the total number and species of plants growing in the feeding grounds were determined. The nutritional value of the pasture sward in the feeding grounds was expressed as the fodder value score (FVS) proposed by Filipek [42]. The fodder value score is an indicator of the nutritional value, yield, and chemical composition of plant species. It ranges from 10 (the highest value) to −3 (poisonous plants) [42]. Additionally, based on the results of plant chemical profiles [43,44], an attempt was made to identify phytoncide plants that are important for animal health and meat quality.

### 2.3. Meat Sampling

After reaching an appropriate age and body weight, the animals were shot in October with the consent and supervision of veterinary services in order to obtain meat for consumption. Ethical review and approval were waived in this study due to the fact that the experimental material was commercially obtained from registered farms rearing red deer, the slaughter of which resulted from the production cycle on the farm and, according to the European Union and Polish legislation (Journal of Laws 2015; Item 266—Act of 15 January on the Protection of Animals Used for Scientific Purposes), is treated as a routine agricultural activity. All post-slaughter procedures (i.e., evisceration, skinning, veterinary inspection) were conducted in an authorized processing plant. The carcasses were cooled at a temperature of 4 °C. The material for the study consisted of samples of *Longissimus lumborum* (LL) and *Musculus semimembranosus* (SM) muscles. Muscles were dissected at 48 h after the slaughter from 36 carcasses of red deer, divided into 3 groups, with 6 hinds and 6 stags in each, with an equal age proportion of ca. 3 and 4 years. Then, they were individually packaged in polyethylene bags and stored at 20 °C until laboratory analysis.

### 2.4. Chemical Analysis

Fat was determined according to the procedure proposed by Folch [45]. Briefly, a 5-g sample of meat was homogenized with 5 mL of methanol, extracted using an automated Soxhlet extractor (Soxtec Avanti, Tecator), and converted into fatty acid methyl esters (FAME). Fatty acids were saponified and esterified in accordance with the PN-ISO 1444:2000 standard [46]. A gas chromatograph (Varian 450-GC with an FID detector) equipped with a flame ionization detector and fitted with a Select™ Biodiesel for FAME capillary column (30 m × 0.32 mm internal diameter and 0.52 µm film thickness, Shinwa Inc.) was used for the separation and quantification of fatty acid methyl esters. The detailed laboratory procedures were previously described by Kilar and Kasprzyk et al. [47]. All samples were analyzed in triplicate. The FA content was expressed in g/100 g of muscle tissue calculated from total intramuscular fat (IMF) using a conversion factor of (*F* _CON_) 0.721 [47].

The results were expressed as g/100 g of total identified fatty acids. The cholesterol content (expressed as mg/100 g of fresh meat) was determined with a gas chromatograph (GC—2010 Shimadzu, Kyoto, Japan). Details of the methodology are described in the previous work [47]. Fatty acid methyl esters were analyzed by gas chromatography (Varian 450-GC with an FID detector) using a fused silica capillary column (30 m × 0.32 mm × 0.52 µm film thickness, Select™ Biodiesel, Shinwa Inc., Nagoya, Japan), following the instructions from a previously published paper [47]. The cholesterol content was expressed as mg/100 g of fresh meat. Using the content of particular fatty acids (FA), the following groups of fatty acids were determined: SFAs—saturated fatty acids, UFAs—unsaturated fatty acids, MUFAs—monounsaturated fatty acids, PUFAs—polyunsaturated fatty acids, PUFA n-6—polyunsaturated fatty acid n-6, and PUFA n-3—polyunsaturated fatty acid n-3. Their ratios (SFA/UFA; MUFA/SFA; PUFA/SFA; PUFA n-6/PUFA n-3) were calculated as well. The atherogenic index (AI), thrombogenic index (TI), ratio of hypo- and hypercholesterolemic fatty acids (h/H), nutritional value (NV), health-promoting index (HPI), cholesterol index (CI), cholesterol–saturated fat index (CSI), and activities of Δ9-desaturase C16, Δ9-desaturase C18, and elongase, hypercholesterolemic fatty acids (OFA), and desirable fatty acids (DFA) were calculated as described before [47].

The concentrations of cadmium (Cd) and lead (Pb) were analyzed on an inductively coupled plasma mass spectrometer (ICP Mass Spectrometer Varian MS-820) according to the Polish Standard, as described before [48].

### 2.5. Statistical Analysis

Data were statistically processed using the Statistica software (v. 13.3, TIBCO Software Inc., Palo Alto, CA, USA). The normality of the data distribution was evaluated using the Shapiro–Wilk test; next, the following mathematical model of one-way analysis of variance (ANOVA) was used:

Yij = μ + FGi + eij, in which Yij was the analyzed variable, μ was the overall mean, FG was the effect of the feeding grounds (i = 1, 2 or 3), and e was the residual error. Duncan’s post hoc test was performed to compare particular means. The results were expressed as the mean ± standard error. Differences were considered significant at *p* < 0.01 and *p* < 0.05, whereas 0.05 ≤ *p* < 0.10 values indicated a tendency.

## 3. Results

**Figure 1:**
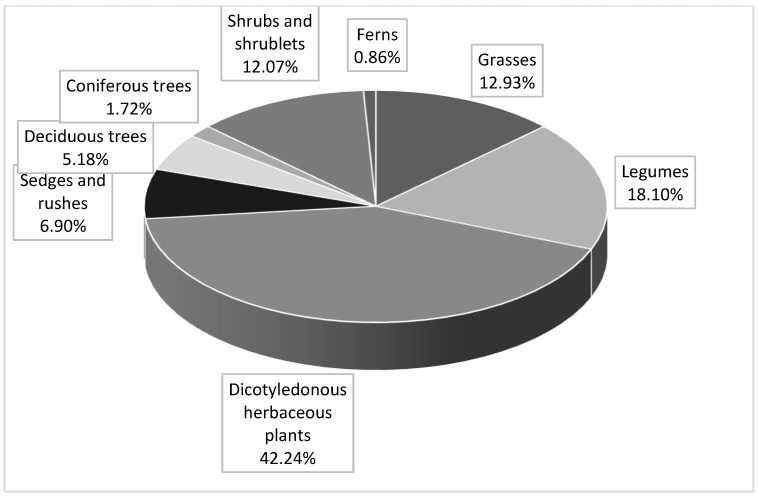
Structure of plant groups in the organic feeding grounds.

**Figure 2:**
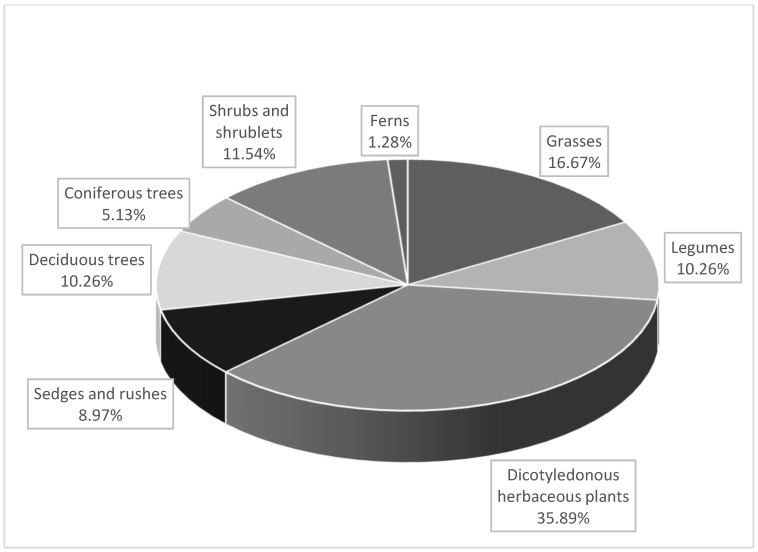
Structure of plant groups in the conventional farm feeding grounds.

The results of the floristic study are presented in Table 1. The organic feeding ground was characterized by the presence of 116 species of vascular plants, with 60 species exhibiting phytoncidal properties. In turn, 78 plant species, including 48 phytoncide plants, were identified in the conventional feeding ground. Dicotyledonous herbaceous plants dominated in both the organic (Figure 1) and the conventional (Figure 2) feeding grounds. As shown in Table 2, the pasture sward in both farm feeding grounds had relatively low nutritional value, as the majority of plants were characterized by FVS below 5. The forest feeding ground was dominated by the following trees: *Pinus sylvestris* L., *Quercus robur* L., *Carpinus betulus* L., *Betula alba* L., *Salix caprea* L., as well as shrubs and shrublets. *Rhanus frangula* L., *Vaccinum vitis-idaea* L., *Sambucus nigra* L., *Calluna vulgaris* (L.) *Hull*, *Rubus idaeus* L., *Corylus avellana* L., *Rubus plicatus W.et N.*, *Juniperus communis* L. Spośród bobowatych licznie występowały *Trifolium pratense* L., *Trifolium arvense* L., *Trifolium repens* L., *Vicia hirsute*, *Vicia cracca*, and *Vicia angustifolia* L. were the predominant legume species in this feeding ground. Additionally, there were ferns, sedges, and rushes.

**Figure 3:**
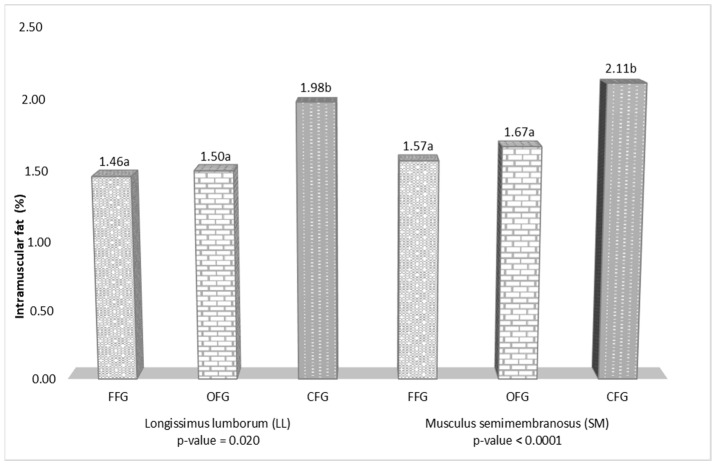
Content of intramuscular fat (%) in the meat of red deer from the three feeding grounds (FFG—forest feeding ground; OFG—organic feeding ground; CFG—conventional feeding ground). For each muscle, means in the same row sharing different letters are significantly different.

The content of intramuscular fat in the LL and SM muscles is presented in Figure 3. In both muscles, the content of intramuscular fat, i.e., 1.98% in LL and 2.11% in SM, was significantly higher in the samples from the conventional farm feeding grounds.

**Figure 4:**
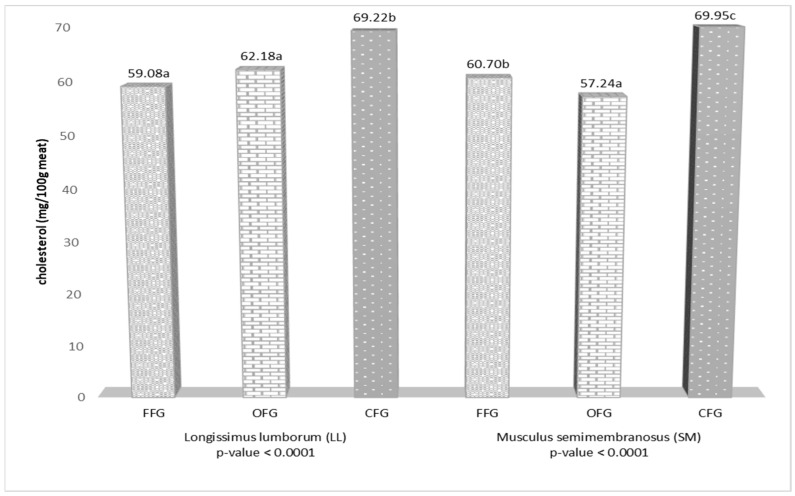
Content of cholesterol (mg/100 g meat) in the meat of red deer from the three feeding grounds (FFG—forest feeding ground; OFG—organic feeding ground; CFG—conventional feeding ground). For each muscle, means in the same row sharing different letters are significantly different.

The cholesterol content (mg/100 g meat) in the LL muscle was 59.08 in the meat from the forest feeding grounds, 62.18 in the meat from the organic farm, and 69.22 in the meat from the conventional system. The conventional farm feeding grounds had a significant (*p* ≤ 0.0001) effect on the cholesterol content in LL (Figure 4). The organic farm system contributed to a significant (*p* ≤ 0.0001) decrease in the cholesterol level in the red deer SM muscle by 3.46 mg/100 g and 12.71 mg/100 g, in comparison with samples from the forest and conventional farm feeding grounds, respectively. Significant differences were also found between samples from the forest feeding grounds and the conventional farm (Figure 4).

The fatty acid profile in the analyzed red deer meat is presented in Table 3. The fatty acid content in LL and SM depended on the feeding ground type, but the impact of this factor varied. In terms of the content of saturated fatty acids in LL, the meat of the red deer from the forest feeding grounds had a significantly lower level of C10:0, C15:0, and C24:0. The meat from the organic farm was characterized by the presence of all determined fatty acids, with significantly lower content of C13:0, C14:0, and C20:0. In turn, the meat from the conventional feeding ground did not contain C22:0 but exhibited significantly higher levels of C10:0, C12:0, C13:0, C15:0, and C24:0. The LL and SM muscles had higher content of C14:1, C15:1, C18:1n9t, and C24:1n9 in the group of animals from the organic farm and C16:1, C17:1, and C18:1n9c in the group of red deer from the conventional feeding grounds.

The analysis of the polyunsaturated FA profile revealed that, regardless of the feeding ground type, LL and SM had the highest amounts of C18:2n6c (LA) and C18:3n3 (ALA), with significant differences. The LL and SM muscles of the red deer from the organic farm contained considerably higher amounts of C18:2n6t, C:18:2c9t11 (CLA), C20:2n6, C22:2n6, C20:4n6 (AA), and C20:5n3 (EPA). In turn, the LL and SM muscles in the group from the forest feeding grounds showed significantly higher (*p* ≤ 0.001) levels of C18:3n6 (GLA), C20:3n6, C20:3n3, and C22:6n3 (DHA).

The SFA sum (g/100 g FA) ranged from 44.21 to 46.81 in LL and from 45.21 to 47.12 in SM (Table 4). In both muscles, the feeding ground type had no significant effect on the sum of UFAs and OFAs, but significant differences were noted in the sum of MUFAs, PUFAs, n-6 PUFAs, and n-3 PUFAs. Higher content of n-6 PUFAs and n-3 PUFAs was determined in the meat of red deer from the forest feeding grounds, in comparison with samples from the organic and conventional systems. The PUFA/SFA ratio in LL and SM was significantly higher in the samples from the forest feeding grounds. The n-6 PUFA/n-3 PUFA ratio in the LL and SM muscles was significantly (*p* ≤ 0.0001) higher in the meat of red deer from the conventional farm system. The analysis of the nutraceutical indices presented in Table 4 revealed significant differences in the TI values and Δ9-desaturase C16 activity, which were higher in the muscles of animals from the conventional farm versus the organic farm and the forest feeding grounds. The DHA+EPA index in the LL and SM muscles had the highest value (*p* ≤ 0.0001) in the meat of red deer from the forest feeding grounds. The health-promoting index (HPI) of the LL muscle in the meat of red deer from the organic system was significantly (*p* = 0.018) higher than in the other red deer groups. The CI and CSI indices were significantly (*p* ≤ 0.0001) higher in the meat of red deer from the organic farm and the forest feeding grounds.

Table 5 shows the FAO and EU recommendations for the levels of fat and FAs as an energy source in the adult human diet [49,50], as well as the cholesterol intake [51]. The consumption of 100 g of fresh red deer meat provided the highest coverage of the recommended of cholesterol intake in a 2000 kcal daily diet (LL from 19.66 to 23.08%; SM from 19.00 to 23.01%) and EPA+DHA (LL from 11.60 to 21.63%; SM from 13.84 to 24.40%), followed by n-3 PUFAs (on average: LL from 5.06 to 6.07%; SM from 5.06 to 6.19%), fat (on average: LL from 2.60 to 3.52%; SM from 2.81 to 3.75%), n-6 PUFAs (on average: LL from 1.70 to 1.90%; SM from 1.69 to 2.20%), PUFAs (on average: LL from 1.59 to 1.66%; SM from 1.62 to 1.80%), and MUFAs (on average: LL from 0.82 to 1.29%; SM from 0.86 to 1.33%). As shown by the analysis, the meat of the red deer from the forest feeding grounds had better dietary properties, similar to the meat of the red deer from the organic farm, which had only slightly lower values.

**Figure 5:**
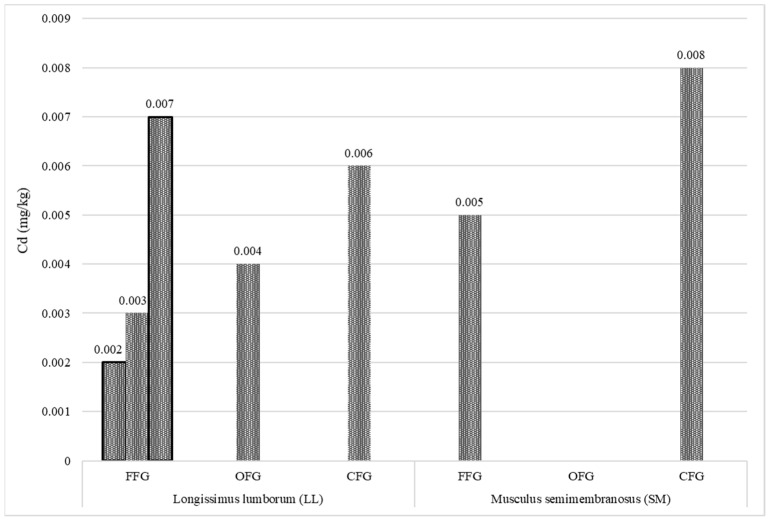
Number of Cd-containing meat samples (mg/kg) collected from the analyzed feeding grounds (FFG—forest feeding ground; OFG—organic feeding ground; CFG—conventional feeding ground).

**Figure 6:**
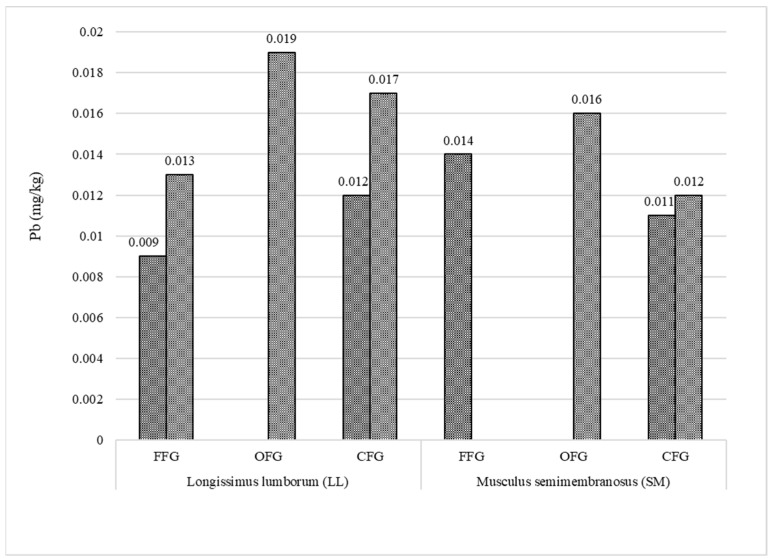
Number of Pb-containing meat samples (mg/kg) collected from the analyzed feeding grounds (FFG—forest feeding ground; OFG—organic feeding ground; CFG—conventional feeding ground).

Cadmium (Figure 5) was detected in five samples of the LL muscle and only in two SM samples. The greatest number of cadmium-containing LL muscle samples was found in the group of the red deer from the forest feeding grounds. No cadmium was detected in any SM muscle sample from the organic farm system. The lowest cadmium content (0.002 mg/kg) was determined in LL from the red deer kept in the forest feeding grounds, while the highest level (0.008 mg/kg) was found in the SM muscle of the animals from the conventional farm feeding grounds. The results of the analysis of the content of lead in the meat are presented in Figure 6. Lead was present in the LL and SM muscles of the red deer from all three feeding ground types. As in the case of cadmium, lead was present in a larger amount of LL muscle samples. The highest lead content was recorded in both muscles (LL—0.019 mg/kg and SM—0.016 mg/kg) sampled from animals kept on the organic farm.

## 4. Discussion

The amount and nutritional value of lipids in meat are important for human nutrition, as they determine the choice and quantity of the product to be consumed, especially given the strong contemporary shift towards the health-enhancing function of food. Due to the focus on health and the concerns about meat safety, consumers are increasingly searching for organic products [2,3,7,8,9] produced in compliance with the principles of natural nutrition, a high level of animal welfare and traceability, and ethical rules of animal breeding [3,36,37,52]. The beneficial impact of organic breeding systems on the nutritional value of meat lipids has been proven in studies on beef [4,53,54,55,56,57], lamb [58], fallow deer [47], rabbits [59], and broiler chickens [60]. In the present study consisting of a comparison of the lipid properties of meat from red deer fed in accordance with different strategies, we intended to provide breeders, consumers, and researchers with information on the health status mainly of meat from organic farm feeding grounds.

The nutritional value of animal meat is largely influenced by nutrition [10,61]. In red deer, the fodder consumed between spring and autumn has paramount importance in this regard. The abundance of forest vegetation and agricultural crops contributes to a richer fatty acid profile and a more pronounced aroma in the meat of free-living red deer [19,23,28]. In this period, the main deer fodder comprises dicotyledonous herbaceous plants, grasses, legumes, sedges, and rushes (up to 80%). It is supplemented by shrublets and mushrooms, as well as the bark, shoots, leaves, and fruits of trees and shrubs [62]. As reported by Estevez et al. [63], plants consumed by red deer from spring to autumn contain elevated content of Ca, K, Mg, Co, Se, Fe, and Zn. In turn, Enri et al. [64] found that *Salix caprea* leaves exhibited high concentrations of C18:2n6, C18:3n6, and C18:3n3, which are regarded as precursors of lipids in the metabolism of ruminants for the synthesis of FAs, exerting a beneficial effect on human health. As reported by Krupka [62], depending on the region of Poland, the food for free-living red deer may comprise approximately 130 species of dicotyledonous plants, 50 species of grasses, 25 species of trees, 40 species of shrubs and shrublets, and 35 species of fungi. The phytoncide richness of such feeding grounds for free-living red deer is of great importance for disease prevention and self-medication among animals [65,66]. The present study indicated that the limitations of feeding grounds for farmed red deer reduced the plant species diversity (Table 1). Despite the presence of plant groups that are necessary for animals in both feeding grounds, the organic feeding ground exhibited a greater diversity of species and a higher number of phytoncide plants. It comprised a greater number of herbaceous dicotyledonous and legume species, as well as shrubs and shrublets, which are especially valuable in red deer nutrition.

The content of fat is an important parameter in the assessment of meat quality. The physiological functions of fat have a considerable influence on human health. Excessive amounts and an imbalance in the composition of total dietary fat lead to dangerous diseases, e.g., cancer, obesity, type 2 diabetes, neurological diseases, and cardiovascular diseases [67,68,69,70]. The content of intramuscular fat in the analyzed red deer meat depended on the feeding ground type. Significantly lower content of fat (on average by 0.5 g/100 g meat) in both muscles was determined in the meat samples from the forest and organic farm feeding grounds than in the conventional system. In turn, Rozmaite et al. [23] reported considerably lower fat content in meat of conventionally reared Lithuanian red deer, with a significant (*p* ≤ 0.05) difference in this parameter for the SM muscle. The large differences (from 0.51 to 4.40%) in the fat content in free-living red deer meat reported in various studies [17,28,32,71] should be associated with the biodiversity of the feeding grounds and the ecotypes of animals in the different countries [62]. Significantly lower (by 0.79 g/100 g meat in LL and by 0.63 g/100 g meat in SM) content of intramuscular fat was found in the meat of fallow deer (*Dama dama*) from an organic farm [47]. As shown by Miotello et al. [53], organic beef contained 0.76% of fat (*p* ≤ 0.001) versus 1.31% in conventional meat. Turner et al. [55] found that organic pasture-fed beef had only 31.9 mg of fat per 1 g of muscle. The present study indicated that the consumption of one 100-g serving of fresh red deer meat from the organic farm feeding ground provided 1.50–1.67 g of fat, which accounts for only 1.92–3.79% of the maximum daily recommendations (Table 5). Meat from red deer reared in organic feeding grounds is a natural, low-calorie food of the highest quality. The analyses revealed a significant (*p* ≤ 0.0001) relationship between the cholesterol content in the red deer meat and the feeding ground type. A particularly favorable effect of the organic feeding grounds on the cholesterol content was noted in the SM muscle, where the amount of this component was reduced by over 22%, compared with that in the meat from the conventional system. Similarly, the cholesterol levels were also reduced (by 11.1%) in the LL muscle from red deer kept in the organic farming conditions in comparison to the conventional variant. Lower cholesterol content in both muscles was noted in the samples from the forest feeding grounds than in the meat from the conventional farm. As reported by Strazdina et al. [72], meat of red deer reared on an organic farm had 19.5 mg% lower cholesterol levels than meat from a conventional farm. Similarly, Ribas-Augusti et al. [4] observed that organic beef had 17% less cholesterol than conventionally produced beef. Polak et al. [27] found that the concentration of cholesterol in the meat of free-living red deer ranged from 73.45 to 94.64 mg/100 g, whereas Quaresma et al. [29] reported an average level of the compound of 55.6 mg/100 g of muscle. The lower cholesterol content in the meat of the organically farmed red deer and the free-living red deer (Figure 2) observed in the present study has a beneficial effect on the health of adults. A 100-g portion of fresh meat of these two groups of red deer contained approximately 57–62 mg of cholesterol, which corresponds to only 20% of the recommended daily intake (Table 5). It should be emphasized that a small amount of cholesterol in the daily diet is desirable, as it is an important component of the cell membrane, brain, and steroidogenic tissue, but its excess may cause coronary artery disease [73].

In addition to the fat content, the fatty acid composition is one of the most important determinants of the quality and pro-health value of meat [1,10,52,61,65]. In ruminants, it is a result of lipid lipogenesis in the adipose tissue, followed by biohydrogenation in the rumen [33,61]. Rumen microorganisms carry out the biohydrogenation of 60 to 90% of unsaturated acids of feed origin. The transformations lead to the generation of saturated FAs, monounsaturated *trans*-As, and CLA. The intensity of biohydrogenation depends mainly on the type of PUFA- and MUFA-containing feed. Roughage is mainly a source of linoleic (C18:2) and linolenic (C18:3) acids, whereas cereal grains are a source of oleic (C18:1) and linoleic (C18:2) acids [57,61,69].

In our study, the meat derived from organically farmed red deer was characterized by lower content of C12:0 and C14:0 in the LL muscle and C12:0 in SM, compared to the muscles of the conventionally reared animals (Table 3). However, no statistically significant differences were found in the total SFA content (Table 4). These results are consistent with the findings reported by Razmaitė et al. [71], who did not observe differences in the content of SFAs between free-living and farmed red deer. In other studies, Razmaitė et al. [17] showed that the SFA content in the skeletal muscles of free-living red deer ranged from 33.56 to 35.62% of total FAs. As reported by Milczarek et al. [18], the SM muscle of free-living red deer contained higher amounts of SFA (47.358%) than the muscle of roe deer (42.688%). Kilar and Kasprzyk [47] found that the organic fallow deer rearing system contributed to a clear decrease in the SFA content in LL, and the differences in SM were significant (*p* ≤ 0.011). Wiklund [74] reported higher levels of SFAs in the *M. longissimus* of pasture-grazed red deer versus animals receiving commercial granulated fodder. As shown by Antunović et al. [58], supplementation of feed concentrate with 13% and 26% of peas had no significant effect on the SFA content in the meat of lambs reared in the organic system. An analysis of the SFA level in beef produced in three fattening systems [54] indicated that the most valuable meat was obtained from animals grazed on an organic pasture. However, steaks produced from this meat were the least appreciated by American beef gourmets.

It is commonly believed that ruminant meat is rich in SFAs [52,56], which was not confirmed in the present study. SFAs accounted for less than 45 and 47% of all FAs present in the muscles of the red deer from the analyzed feeding grounds, and the consumption of a 200-g portion of fresh meat was found to provide 0.94–1.45 g of SFAs, constituting only 2.14–3.26% of the recommended intake. Thus, considering health indications, red deer meat can be an alternative to other types of red meat. Similar conclusions were formulated in studies of fallow deer meat [47]. The most frequent SFAs in the lipid profiles of the analyzed muscles were C16:0 (palmitic acid), C18:0 (stearic acid), and C14:0 (myristic acid). These results are in agreement with those reported by Milczarek et al. [18], Razmaitė et al. [23], Ugarković et al. [28], and Nagy et al. [33]. Although SFAs are considered harmful to health, the potential of myristic acid to increase total serum cholesterol is 4- to 6-fold higher than that of C16:0 [75]. Moreover, not all SFAs pose a health risk: even high content of stearic acid exerts no effect on cholesterol levels, as it is poorly digested and easily desaturated to C18:1 [76].

The proportions of MUFAs and SFAs are mainly affected by ruminal biohydrogenation, which in turn may be influenced by several factors [76]. It was found that the percentage of monounsaturated fatty acids (MUFAs) was higher in the muscles of the conventionally farmed red deer than in the case of animals from the forest feeding grounds, due to the higher concentrations of C16:1 and C17:1. It was also similar to the value determined for the muscles of the red deer from the organic system. Investigations conducted by Karac et al. [77] revealed higher levels of C14:1, C16:1, and C17:1 in concentrate-fed lambs than in pasture-fed lambs. The activity of Δ^9^ sterol-CoA desaturase (Δ^9^ C16) was higher in the muscles of red deer from the conventional farm than in the animals from the organic farm and forest feeding grounds (Table 4). Typically, the concentration of C16:1 acid increases with the increasing activity of the Δ^9^ desaturase enzyme, which synthesizes palmitoleic acid from palmitic acid. The present study showed that oleic acid (C18:1) was the dominant monounsaturated acid, which is in line with the results reported by Ugarković et al. [28], Revilla et al. [57], Kilar and Kasprzyk [47], and Cividini et al. [78]. Elevated MUFA content is extremely desirable and recommended by nutritionists. Increased consumption of MUFAs reduces the level of total plasma cholesterol and the LDL fraction and does not increase the level of triglycerides. It has been evidenced that MUFAs, as a dietary component, play a protective role in atherosclerosis prophylaxis [61]. Additionally, the concentration of C16:1 and C18:1 MUFAs has been positively correlated with flavor, flavor preference, and overall acceptability [54,79].

Notably, ruminant meat is one of the natural sources that is rich in CLA isomers, especially 18:2c9t11 [56,80,81]. In the present study, the CLA concentration in the meat from the red deer reared in the organic farm system was over 17 and 40% higher in LL and SM than in the muscles of animals from the conventional farm. In beef from grazing cattle, the proportion of the *cis*-9 *trans*-11 CLA isomer was over double that from the feedlot system [82]. The CLA content in ruminant-derived products depends on the supply of LA [33] and is a result of the endogenous desaturation of trans-vaccinic acid by the Δ9-desaturase enzyme [61]. Legumes and grasses are a rich source of linoleic acid (LA), which is contained in chloroplasts. These organelles are generally engulfed by rumen protozoa and thus act as a source of “protected fatty acids” [82]. Additionally, the increase in the CLA concentration in the meat from the organic farm and forest feeding grounds observed in the present study may be associated with the higher fiber content, compared with the conventional system; consequently, this may enhance the growth and activity of bacteria (*Butyrivibrio fibrisolvens*), inhibiting reductase enzymes that catalyze the conversion of vaccenic acid to stearic acid [61]. Many studies confirm the thesis that diets based on such a variety of grasses, legumes, and herbaceous plants as that available to animals in organic feeding grounds increase the total CLA content in meat [33,56,61,78,80,83]. In the present study, the CLA content in the LL and SM muscles of red deer from the organic farm was approximately 1.69 and 1.92 g/100 g, respectively; it was considerably greater than in meat from the conventionally fed animals, probably due to the ground being characterized by poorer plant species cover. Higher levels of rumen acid in organically farmed animals may increase the value of their meat and products due to its role in the prevention and even treatment of diabetes, obesity, osteoporosis, and some cancers [13,19,52]. A substantially lower concentration of CLA than that in the present study was found in the meat of cattle fed with concentrate and grass, i.e., 5.6 and 6.7 mg/100 g, respectively [83]. As shown by Bautista-Martine et al. [82], there are differences in CLA concentrations even in grazing-based systems. This is related to the variability of C18:3n3, C18:2n6, and C16:0 depending on the grass species, growth phase, light intensity, temperature, and agroecological factors.

From the point of view of the physiology of human nutrition, long-chain polyunsaturated FAs (LC-PUFAs), e.g., linoleic acid LA (n-6 family) and alpha-linolenic acid ALA (n-3 family), are especially important. They constitute a pool of essential unsaturated FAs and are absolutely necessary for the development and proper functioning of the human organism. Both the n-3 and n-6 PUFA families compete for the same enzymes in the processes of their elongation and desaturation [69]. In the present study, the red deer from the organic feeding grounds grazed on a pasture with many different plant species, which were also more numerous than in the conventional feeding grounds. The type and quality of pasture and forage are assumed to affect the proportion of the 18:3 fatty acid in meat [70,81]. The most abundant FAs in grasses, legumes, and forbs are ALA (approximately 62%), LA, and palmitic acid, which together account for up to 93% of all FAs [84]. White and red clover, which were part of the species cover in the organic feeding ground, have higher PUFA content than that in a conventional pasture dominated by *Lolium perenne* [85]. As a consequence of the higher intake of ALA, the intramuscular fat of both muscles of animals from the organic farm contained higher amounts of polyunsaturated acids EPA and DHA (*p* ≤ 0.01) than the meat of animals from the conventional farm system (Table 3). These observations confirm the higher dietetic value of these muscles. This differentiation results from the different effects of the utilized fatty acid sources on the populations of microorganisms participating in the biohydrogenation process. Moreover, the lipolytic activity of the enzymes contained in legumes, e.g., polyphenol oxidase, contributes to an increase in the transfer of polyunsaturated fatty acids from feed to meat, mainly through the reduction of lipolysis, resulting in greater protection of PUFAs across the rumen [85]. As suggested in the literature [33,56,61,78,80,83], the use of appropriate components of the feed for ruminants can modulate fermentation processes, aimed at the production of fatty compounds characterized by biological activity. Additionally, the effects of the different animal IMF accumulation might also have altered the fatty acid profile. The higher concentrations of EPA and DHA may result from the desaturation and elongation of FAs in the tissue [56,83]. Eicosapentaenoic fatty acid (EPA and docosahexaenoic acid (DHA) can be synthesized in the mammalian organism from α-linolenic n-3 acid (ALA), whereas acids from the n-6 group, such as GLA and AA, derive from linoleic acid (LA) [70]. This is extremely important, as the consumption of n-3 LC-PUFAs, e.g., EPA and DHA, exerts a positive effect on health and reduces the prevalence of many diseases [27,69,70,86,87]. Notably, the consumption of 100 g of meat from animals reared in the organic farm system was found to provide from 40 mg to 48 mg of EPA and DHA, which constitutes 22–26% of the recommended daily intake (Table 5). Similar to the present findings, a higher concentration of EPA and DHA was determined by Kamihiro et al. [56] in steaks from meat produced in the organic cattle breeding system. Strazdina et al. [23] reported higher content of DHA in the meat of farm red deer and almost the same level of EPA in the meat of free-living and farm red deer. As shown by Milczarek et al. [18], the content of both these acids was significantly lower in red deer meat than in roe deer meat. In turn, meat of wild axis deer contained 1.63% of EPA and only 0.19% of DHA [28].

PUFA/SFA ratios are important indicators of the overall fatty acid quality in meat. Such data can be extremely useful for the early evaluation and monitoring of nutritional strategies targeted at the improvement of the nutraceutical composition of meat [65,88]. A higher value of this indicator was recorded in the meat of red deer from the organic farm and the forest feeding grounds. Despite these differences, the values were within the recommended limits (min. 0.4) [67]. The present study indicated that the greater richness of the forest and organic farm feeding grounds contributed to the higher content of n-6 PUFA and n-3 PUFA. Kamihiro et al. [56] reported a higher level of n-6 PUFA in conventional beef and n-3 PUFA in organic beef. It is generally believed that a concentrate- or cereal-based diet contains higher levels of n-6 PUFA, especially LA, than a pasture-based diet [78]. Consequently, the meat of animals receiving concentrated feed or grains should contain greater amounts of LA than grazing animals. Opposite results were obtained in the present study (Table 3).

The n-6/n-3 ratios and the Al and TI indicators are also used to assess the nutritional value of fat [67]. It was shown in the present study that both muscles sampled from red deer from the organic farm and forest feeding grounds exhibited a significantly better n-6/n-3 ratio (Table 4), which is consistent with the results reported by Purchas et al. [34]. Higher values (from 2.74 to 5.04) were reported by Ugarković et al. [28]. In a study conducted by Strazdina et al. [23], the n-6/n-3 ratio was slightly higher in the meat of farmed red deer but did not exceed 2.54. As demonstrated by Gruffat et al. [80], the n-6/n-3 ratio decreased to 1.8 in the meat of lambs fed alfalfa greens and to 1.6 when alfalfa pellets were used. The n-6/n-3 ratio was 2.922 in the organic cattle grazing system and ranged from 7.166 to 8.046 in two conventional systems [57]. A low n-6/n-3 ratio is desirable as a means of reduction of the risk of many high-frequency chronic diseases in both Western and developing countries [61,65,80]. Although the role of the ratio of n-6 and n-3 acids in the human diet has not been clearly elucidated, the recommended n-6 PUFA/n-3 PUFA ratio should not exceed 4:1 [49]. The specified quantitative proportion as a criterion for proper human nutrition is not as important as suggested, and the role of the ratio in the prevention of many diseases is often unreliable [61]. As proposed by the FAO [49], in the case of n-3 and n-6 fatty acids, it should be assumed that it is more important to achieve an appropriate consumption threshold (absolute amount)—in particular, of ALA and LC n-3 PUFA. It should be noted that the consumption of 100 g of organic red deer meat may provide, on average, 149 mg of LC-PUFA n-6 and 100 mg of LC-PUFA n-3 (Table 5). The consumption of food with a lower AI index may contribute to total cholesterol and LDL-C level reduction in human blood plasma [67]. In our study, the relatively low TI and AI values determined in the muscles of red deer reared in the organic farm indicate that this meat may have beneficial effects on human health in terms of the fatty acid composition.

Cadmium and lead are dangerous elements exerting toxic effects on humans and nature [30,33,89]. Particular attention to the presence of these elements is paid in organic production systems [5,90]. As specified by the applicable law [91,92], the permissible content of these elements in meat is 0.05 mg/kg (cadmium) and 0.10 mg/kg (lead). The present study showed that the analyzed red deer meat contained significantly lower amounts of cadmium and lead, and the meat from the organic system was found to be safer. The greater total number of samples that contained the elements in the group of red deer from the forest feeding grounds can be associated with the larger living area available to these animals. Ciobanu et al. [89] reported that the Cd and Pb accumulation in roe deer meat was related to the type of muscle. The lowest amounts of cadmium and lead were found in *M. triceps brachii* and the highest content was determined in *M. longissimus dorsi*. As shown by Cygan-Szczygielniak [30] in a study of red deer from Kujawsko-Pomorskie Province, the hair and LL muscle of the animals had the highest levels of lead, while the liver and kidneys were characterized by the highest content of cadmium. The content of cadmium and lead in red deer meat determined by Milczarek et al. [18] did not exceed 0.008 mg/kg and 0.03 mg/kg, respectively. Importantly, given the low consumption of venison in Poland [21], the cadmium and lead content in the red deer meat from all the three feeding grounds analyzed in the present study does not pose a threat to consumers.

## 5. Conclusions

UFAs dominated in the meat of the red deer from the three feeding grounds (LL: from 53.19 to 55.79 g/100 g; SM: from 52.88 to 54.80 g/100 g). SFAs constituted from 44.21 to 46.81 g/100 g in LL and from 45.21 to 47.12 in SM, with the largest share of C16:0 and C18:0. C18:1n9c was the dominant MUFA. This study is the first attempt to fill the gap in the knowledge of the nutritional value of meat from red deer reared in the organic farming system. The organic LL and SM were found to have significantly lower amounts of fat and cholesterol than the conventionally produced meat. Interestingly, the cholesterol content in the SM muscle was even lower than in the meat of the red deer from the forest feeding ground. The data obtained in the investigations are important for consumers, as the results help to select food with appropriate dietetic value. Regarding the fatty acids, the differences in the fatty acid composition between the groups are quite small. The low concentration of IMF in the case of FFG and OFG contributed to a high percentage of PUFA content (n-3 PUFA, EPA, and DHA), in comparison to CFG. It may also be supposed that the greater species and phytoncide diversity of plants present in the organic and forest feeding grounds enriched the quantitative profile and increased the content of the desired polyunsaturated FAs, although further detailed chemical studies of plants are necessary in order to confirm this assumption. The meat of the red deer from the forest feeding ground was found to be a rich source of GLA, and especially DHA, which was reflected in the higher value of the important nutraceutical EPA+DHA index. As demonstrated in the present study, the n-6/n-3 FA ratio and the TI values are more beneficial in the meat of OFG and FFG than CFG. These parameters may contribute to the improvement of human health, well-being, and resistance to diseases. In particular, the concentrations of DHA and EPA in the red meat could be more critical in future alternatives for human nutrition. The levels of cadmium (from 0.002 to 0.008 mg/kg) and lead (from 0.009 to 0.019 mg/kg) in the red deer meat were found not to pose a threat to consumers. The present results have great scientific relevance and suggest that deer meat from FFG and ORG can be considered an alternative or complementary source of n-3 PUFAs. In turn, their utilitarian importance is associated with the support of breeders in making decisions about the locations of farms and the choice of red deer nutrition strategies.

## Figures and Tables

**Table 1:** Plant species identified in the red deer farm feeding grounds.

Plant Groups	Plant Species	
	Organic Feeding Ground (OFG)	Conventional Feeding Ground (CFG)
Grasses	*Alopecurus pratensis* L.; *Arrhenatherum elatius* (L.) *P.B. ex J. et C. Presl*; *Agropyron repens* (L.) *P.B.*; *Dactylis glomerata* L.; *Deschampsia caespitosa* (L.) *P.B.*; *Lolium temulentum* L.; *Lolium multiflorum Lam.*; *Lolium perenne* L.; *Poa annua* L.; *Poa pratensis* L.; *Poa trivialis* L.; *Setaria glauca auct*. *; *Setaria viridis* (L.) *P.B*. *; *Festuca rubra* L.; *Festuca arundinacea Schreb*. *	*Agropyron repens* (L.) *P.B*; *Agrostis vulgaris With.*; *Alopecurus pratensis* L.; *Arrhenatherum elatius* (L.) *P.B*; *Corynephorus canescens* (L.) *P.B.*; *Dactylis glomerata* L.; *Deschampsia caspitosa* (L.) *P.B.*; *Echinochloa crus-galli* (L.) *P.B.*; *Festuca rubra* L.; *Lolium perenne* L.; *Phleum pretense* L.; *Poa annua* L.; *Poa pratensis* L.
Legumes	*Lotus corniculatus* L. *; *Trifolium arvense* L. *; *Astragalus glycyphyllos* L.; *Ononis spinosa* L.; *Medicago lupulina* L. *; *Trifolium pratense* L. *; *Trifolium repens* L. *; *Trifolium strepens Cr. = T. aureum Poll*. *; *Vicia cracca* L. *; *Vicia hirsuta* (L.) S.F. Gray *; *Vicia tetrasperma* (L.) Schreber *; *Vicia angustifolia* L. *; *Trifolium hybridum* L.; *Lotus uliginosus* L.; *Medicago lupulina* L.; *Lathyrus pratensis* L.; *Trifolium dubium Sibth.*; *Trifolium fragiferum* L.; *Galega officinalis* L.; *Lotus tenuis Waldst. & Kit. ex Willd.*; *Lupinus luteus* L.	*Trifolium pratense* L. *; *Trifolium repens* L. *; *Vicia tetrasperma* (L.) *Schreb*. *; *Ononis spinosa* L.; *Trifolium arvense* L. *; *Vicia hirsuta* (L.) S.F. Gray *; *Vicia tetrasperma* (L.) *Schreber*; * Medicago lupulina L. *
Dicotyledonous herbaceous plants	*Holosteum umbellatum* L. *; *Myosotis palustris* (L.) Nathorst *; *Moehringia trinervia* (L.) *Clairv*. *; *Oxalis stricta* L. *; *Geranium pusillum* L. **; *Erigeron canadensis* L. *; *Crepis biennis* L. *; *Cerastium vulgatum* L. *; *Matricaria chamomilla* L. **; Myosotis stricta Link *; *Plantago major* L. **; *Plantago lanceolata* L. **; *Prunella vulgaris* L. **; *Ranunculus repens* L. **; Viola arvensis Murray **; *Capsella bursa pastoris*; *Cerastium arvense* L.; *Lychnis flos-cuculi* L.; *Saponaria officinalis* L.; *Matricaria inodora* L. **; *Cardamine pratensis* L. **; *Nuphar lutea*; *Anemone nemorosa* L.; *Urtica dioica* L. *; *Agrimonia eupatoria*; *Alchemilla pastoralis Bus*. **; *Alchemilla xanthochlora Rothm.*; *Potentilla anserine* L. **; *Ranunculus acris* L.; *Stellaria media Vill*. *; *Symphytum officinale* L. **; *Glechoma hederacea* L.; *Lamium album* L.; *Stachys sylvatica* L. *; Valeriana officinalis **; *Bellis perennis*; *Tanacetum vulgare* L. *; *Convallaria majalis* L.; *Geranium pratense* L.; *Linaria vulgaris* L. **; *Solidago virgaurea* L.; *Aruncus dioicus*; *Vinca minor* L. *; *Lythrum salicaria* L.; *Polygonatum multiflorum*; *Angelica sylvestris* L.; *Daucus carota* L.; Taraxacum officinale Weber **; *Achillea millefolium* L. **	*Aegopodium podagraria* L. **; *Alchemilla pastoralis Bus*. **; *Achillea millefolium* L. **; *Centaurea jacea* L. *; *Cirsium arvense* (L.) *Scop.*; *Erigeron annuus* (L.) *Pers*. *; *Erigeron canadensis* L. *; *Equisetum arvense* L. *; *Hypericum perforatum* L. **; *Hieracium pilosella* L. **; *Linaria vulgaris* L. **; *Lysimachia nummularia* L. **; *Plantago major* L. **; *Potentilla anserine* L. **; *Prunella vulgaris* L. **; *Ranunculus bulbosus* L. **; *Rumex acetosa* L. *; *Rumex crispus* L. *; *Silene inflata* (*Salisb.*) *Sm*. *; *Taraxacum officinale Web*. *; *Arctium lappa* L.; *Galeopsis tetrahit* L. *; *Lychnis flos-cuculi* L.; *Geranium pratense* L.; *Vinca minor* L. *; *Cerastium arvense* L.; *Bellis perennis*; *Geum urbanum* L.
Sedges and rushes	*Juncus articulatus* L. *; *Juncus effusus* L.; *Juncus bufonius* L. *; *Luzula campestris* (L.) *DC*. *; *Carex distans* L. *; Carex glauca Murr *; *Carex hirta* L. *; *Carex leporina* L. *	Carex glauca Murr *; *Carex hirta* L. *; *Carex leporina* L. *; *Carex distans* L. *; Juncus bufonius *; *Juncus conglomeratus* L. *; *Luzula campestris* (L.) *DC*. *
Deciduous trees	*Betula alba* L. **; *Salix caprea* L. **; *Carpinus betulus* L.; *Fagus sylvatica* L.; *Quercus robur* L.; *Alnus glutinosa* (L.) *Gaertn.*	*Betula alba* L. **; Prunus domestica *; *Salix aurita* L. **; *Salix caprea* L. **; *Salix cinerea* L. *; *Quercus robur* L. *; Betula pendula ssp. Obscura *; *Alnus glutinosa* (L.) *Gaertn.*
Coniferous trees	*Pinus sylvestris* L. **; *Picea abies* (L.) *H.Karst*	*Pinus sylvestris* L. **; *Picea abies* (L.) *H. Karst*; *Abies alba*; *Picea pungens Engelm.*
Shrubs and shrublets	Rubus laciniatus *; *Rubus idaeus* L. *; *Vaccinium vitis-idaea* L.; *Sambucus nigra* L. **; *Juniperus communis* L. *; *Empetrum nigrum* L.; *Calluna vulgaris* (L.) *Hull*; *Corylus avellana* L. **; *Heuchera micrantha*; *Padus padus*; Frangula alnus **; Thymus serpyllum *; Prunus spinosa **; *Euonymus verrucosus*	*Rubus plicatus W. et N*. *; Rubus nessensis Hall *; *Rubus laciniatus*; *Aruncus dioicus*; *Rubus idaeus* L. *; *Sambucus nigra* L. **; *Vaccinium vitis-idaea* L.; *Crataegus monogyna Jacq.*; *Juniperus communis* L. *
Ferns	*Athyrium filix-femina* L. *	*Athyrium filix-femina* L. *

** phytoncide plants with strong therapeutic and prophylactic properties; * phytoncide plants with moderate and weak therapeutic and prophylactic properties.

**Table 2:** Nutritional value of plants growing in the farm feeding grounds, expressed as a fodder value score (FVS).

FVS	Plant Species	
	Organic Feeding Ground (OFG)	Conventional Feeding Ground (CFG)
10	*Lolium perenne* L.; *Poa pratensis* L.; *Trifolium repens* L.	*Lolium perenne* L.; *Phleum pretense* L.; *Poa pratensis* L.; *Trifolium repens* L.
9	*Alopecurus pratensis* L.; *Arrhenatherum elatius* (L.) *P.B. ex J. et C. Presl*; *Dactylis glomerata* L.; *Lotus corniculatus* L.; *Lolium multiflorum Lam.*; *Trifolium pratense* L.	*Alopecurus pratensis* L.; *Arrhenatherum elatius* (L.) *P.B*; *Dactylis glomerata* L.; *Trifolium pratense* L.
8	*Medicago lupulina* L.; *Poa trivialis* L.	*Poa trivialis* L.
7	*Agropyron repens* (L.) *P.B.*; *Plantago lanceolata* L.; *Alchemilla pastoralis Bus.*	*Agropyron repens* (L.) *P.B*; *Alchemilla pastoralis Bus.*; *Vicia tetrasperma* (L.) *Schreb.*
6	*Achillea millefolium* L.; *Festuca rubra* L.; *Festuca arundinacea Schreb.*; *Poa annua* L.; *Taraxacum officinale Weber*; *Trifolium strepens Cr. = T. aureum Poll.*; *Vicia cracca* L.	*Agrostis vulgaris With.*; *Achillea millefolium* L.; *Festuca rubra* L.; *Poa annua* L.; *Taraxacum officinale Weber*
5	*Alchemilla xanthochlora Rothm.*; *Tragopogon pratensis* L.	
4	*Cerastium vulgatum* L.; *Cerastium arvense* L.; *Daucus carota* L.	*Rumex acetosa* L.; *Cerastium arvense* L.
3	*Deschampsia caespitosa* (L.) *P.B.*	*Centaurea jacea* L.; *Echinochloa crus-galli* (L.) *P.B.*; *Deschampsia caspitosa* (L.) *P.B.*
2	*Myosotis stricta Link*; *Plantago major* L.; *Prunella vulgaris* L.; *Ranunculus repens* L.; *Stellaria media Vill.*; *Carex distans* L.; *Carex glauca Murr*; *Carex hirta* L.; *Carex leporina* L.; *Geranium pratense* L.; *Myosotis palustris* (L.) *Nathorst*	*Carex distans* L.; *Carex glauca Murr*; *Carex hirta* L.; *Carex leporina* L.; *Hieracium pilosella* L.; *Lysimachia nummularia* L.; *Plantago major* L.; *Prunella vulgaris* L.; *Rumex crispus* L.; *Geranium pratense* L.; *Hypericum perforatum* L.
1	*Juncus articulatus* L.; *Luzula campestris* (L.) *DC.*; *Lychnis flos-cuculi* L.; *Persicaria amphibia* (L.) *Delarbre*; *Potentilla anserine* L.; *Glechoma hederacea* L.; *Valeriana officinalis*; *Bellis perennis*; *Juncus effusus* L.	*Corynephorus canescens* (L.) *P.B.*; *Luzula campestris* (L.) *DC.*; *Potentilla anserine* L.; *Lychnis flos-cuculi* L.; *Bellis perennis*; *Ranunculus acris* L.
0	*Crepis biennis* L.; *Erigeron canadensis* L.; *Geranium pusillum* L.; *Holosteum umbellatum* L.; *Juncus bufonius* L.; *Lolium temulentum* L.; *Matricaria chamomilla* L.; *Matricaria inodora* L.; *Moehringia trinervia* (L.) *Clairv.*; *Oxalis stricta* L.; *Sagina procumbens* L.; *Setaria glauca auct.*; *Setaria viridis* (L.) *P.B.*; *Trifolium arvense* L.; *Vicia hirsuta* (L.) *S.F. Gray*; *Vicia tetrasperma* (L.) *Schreber*; *Vicia angustifolia* L.; *Viola arvensis Murray*; *Nuphar lutea*; *Corydalis cava*; *Barbarea vulgaris*; *Capsella bursa pastoris*; *Saponaria officinalis* L.; *Urtica dioica* L.; *Agrimonia eupatoria*; *Geum urbanum* L.; *Astragalus glycyphyllos*; *Ononis spinosa* L.; *Impatiens noli-tangere*; *Lythrum salicaria* L.; *Angelica sylvestris* L.; *Symphytum officinale* L.; *Lamium album* L.; *Stachys sylvatica* L.; *Tripleurospermum maritimum* L.; *Convallaria majalis* L.; *Polygonatum multiflorum*; *Linaria vulgaris* L.; *Solidago virgaurea* L.; *Arctium lappa* L.; *Aruncus dioicus*; *Vinca minor* L.	*Aegopodium podagraria* L.; *Cirsium arvense* (L.) *Scop.*; *Erigeron annuus* (L.) *Pers.*; *Erigeron canadensis* L.; *Erigeron canadensis* L.; *Galeopsis tetrahit* L.; *Hypericum perforatum* L.; *Juncus bufonius*; *Juncus conglomeratus* L.; *Linaria vulgaris* L.; *Ranunculus bulbosus* L.; *Silene inflata* (*Salisb.*) *Sm.*; *Aruncus dioicus*; *Arctium lappa* L.; *Lychnis flos-cuculi* L.; *Trifolium arvense* L.; *Vicia hirsuta* (L.) *S.F. Gray*
From −1 to −3	*Cardamine pratensis* L.; *Anemone nemorosa* L.; *Ranunculus acris* L.; *Tanacetum vulgare* L.	

**Table 3:** Fatty acid profile (g/100 g FA) in red deer meat sampled from the three feeding grounds.

Fatty Acids	*Longissimus Lumborum* (LL)							*Musculus Semimembranosus* (SM)						
	FFG		OFG		CFG		*p*-Value	FFG		OFG		CFG		*p*-Value
	$\bar{X}$	SE	$\bar{X}$	SE	$\bar{X}$	SE		$\bar{X}$	SE	$\bar{X}$	SE	$\bar{X}$	SE	
C8:0	nd	-	nd	-	0.15	0.034	-	nd	-	nd	-	0.18	0.041	-
C10:0	0.07a	0.015	0.13b	0.059	0.18b	0.061	<0.0001	0.07a	0.016	0.09a	0.023	0.17b	0.036	<0.0001
C12:0	0.19a	0.078	0.19a	0.056	0.31b	0.067	<0.0001	0.20a	0.045	0.19a	0.062	0.32b	0.082	<0.0001
C13:0	0.14b	0.021	0.08a	0.029	0.15b	0.037	<0.0001	0.17b	0.008	0.06a	0.011	0.17b	0.049	<0.0001
C14:0	3.56c	0.788	2.52a	0.371	3.13b	0.726	0.002	2.98	0.697	3.53	0.942	2.87	0.656	0.102
C15:0	0.29a	0.062	0.50b	0.280	0.77c	0.146	<0.0001	0.29a	0.064	0.47b	0.264	0.70c	0.193	<0.0001
C16:0	20.79	1.606	20.70	0.843	20.92	2.212	0.950	21.70	1.039	21.64	1.289	21.65	3.005	0.996
C17:0	0.59	0.287	0.56	0.264	0.76	0.174	0.124	0.58	0.276	0.46	0.236	0.69	0.167	0.065
C18:0	18.63	1.590	18.51	1.165	19.52	1.371	0.164	18.75	2.365	17.56	1.663	19.47	1.520	0.057
C20:0	0.28b	0.085	0.16a	0.114	0.17a	0.062	0.005	0.25	0.083	0.17	0.092	0.20	0.076	0.091
C21:0	nd	-	0.69	0.274	0.59	0.296	0.388	nd	-	0.70	0.129	0.55	0.268	0.107
C22:0	0.07	0.012	0.08	0.018	nd	-	0.294	0.14a	0.052	0.22b	0.022	nd	-	<0.0001
C24:0	0.08a	0.014	0.11a	0.031	0.16b	0.038	<0.0001	0.08a	0.021	0.17b	0.033	0.15b	0.029	<0.0001
C14:1	2.89	0.426	3.21	0.528	2.92	0.585	0.251	3.33	0.659	3.79	1.045	3.04	0.490	0.065
C15:1	0.15	0.015	0.80	1.101	0.44	0.230	0.064	0.14	0.021	0.57	0.798	0.42	0.250	0.102
C16:1	2.99a	0.556	3.32a	0.620	6.14b	1.825	<0.0001	2.77a	0.520	3.36b	0.552	5.79c	1.653	<0.0001
C17:1	0.09a	0.060	0.14a	0.114	0.28b	0.130	<0.0001	0.13	0.074	0.22	0.274	0.28	0.119	0.128
C18:1n9c	21.83	3.418	22.35	2.882	23.64	3.602	0.396	21.69	3.130	22.82	3.937	23.02	4.334	0.662
C18:1n9t	0.27a	0.065	0.54b	0.251	0.21a	0.061	<0.0001	0.29a	0.062	0.60b	0.222	0.21a	0.057	<0.0001
C20:1	0.20a	0.079	0.41b	0.137	0.22a	0.104	<0.0001	0.20b	0.060	0.19b	0.088	0.12a	0.043	0.007
C24:1n9	0.30a	0.037	0.38b	0.047	nd	-	<0.0001	0.26a	0.067	0.43b	0.049	nd	-	<0.0001
C18:2n6c (LA)	9.26b	0.809	7.98a	0.987	8.01a	1.285	0.007	9.25b	1.039	6.40a	0.831	9.09b	1.122	<0.0001
C18:2n6t	0.20a	0.050	0.44c	0.148	0.31b	0.061	<0.0001	0.18a	0.053	0.50c	0.214	0.28b	0.055	<0.0001
C18:2c9t11 (CLA)	1.57ab	0.179	1.69b	0.101	1.44a	0.088	<0.0001	1.71b	0.183	1.92c	0.108	1.37a	0.042	0.035
C20:2n6	nd	-	0.21b	0.029	0.09a	0.033	<0.0001	nd	-	0.16b	0.022	0.12a	0.044	0.005
C22:2n6	nd	-	0.28b	0.047	0.21a	0.074	0.014	nd	-	0.33b	0.028	0.18a	0.056	<0.0001
C18:3n6 (GLA)	2.25c	0.221	1.67b	0.181	1.18a	0.079	<0.0001	2.30c	0.137	1.38b	0.063	1.21a	0.100	<0.0001
C18:3n3 (ALA)	4.45b	0.619	4.91c	0.921	3.93a	0.374	0.005	3.66b	1.039	4.11c	0.599	3.31a	0.341	0.009
C20:3n6	1.48c	0.135	0.82b	0.734	0.23a	0.145	<0.0001	1.56b	0.236	1.55b	0.529	0.18a	0.099	<0.0001
C20:3n3	0.61b	0.299	0.57b	0.221	0.30a	0.068	0.003	0.65c	0.118	0.47b	0.156	0.31a	0.035	<0.0001
C20:4n6 (AA)	1.61a	0.203	2.43b	0.044	1.61a	0.038	<0.0001	1.36a	0.083	1.98c	0.071	1.69b	0.032	<0.0001
C20:5n3 (EPA)	1.52b	0.106	1.69b	0.180	0.84a	0.122	<0.0001	1.70b	0.195	1.82b	0.078	0.88a	0.368	<0.0001
C22:6n3 (DHA)	3.59c	0.100	1.96b	0.084	1.18a	0.079	<0.0001	3.64c	0.125	2.13b	0.104	1.39a	0.055	<0.0001

nd = not detected. The different letters next to the mean value in the same row indicate significant differences between these values for each muscle. FFG—forest feeding ground; OFG—organic feeding ground; CFG—conventional feeding ground.

**Table 4:** Sum of FA groups (g/100 g FA), proportions of fatty acids, and nutraceutical indices of lipids in red deer meat sampled from the three feeding grounds.

Specification	*Longissimus Lumborum* (LL)							*Musculus Semimembranosus* (SM)						
	FFG		OFG		CFG		*p*-Value	FFG		OFG		CFG		*p*-Value
	$\bar{X}$	SE	$\bar{X}$	SE	$\bar{X}$	SE		$\bar{X}$	SE	$\bar{X}$	SE	$\bar{X}$	SE	
**Sums of FA Groups**														
SFA	44.68	2.870	44.21	2.220	46.81	2.886	0.053	45.21	3.197	45.26	2.695	47.12	3.952	0.288
UFA	55.32	2.870	55.79	2.220	53.19	2.886	0.053	54.80	3.197	54.74	2.695	52.88	3.952	0.288
MUFA	28.80a	3.192	31.14ab	2.926	33.85b	2.954	0.001	28.81a	3.339	31.97b	3.268	32.87b	4.295	0.026
PUFA	26.52b	1.016	24.65b	1.658	19.34a	1.651	<0.0001	25.99b	1.971	22.77ab	1.301	20.01a	1.263	<0.0001
PUFA n-6	14.80b	1.101	13.83ab	1.036	11.65a	1.403	<0.0001	14.64b	1.040	12.31a	0.933	12.74a	1.133	<0.0001
PUFA n-3	10.16b	0.759	9.13b	0.983	6.25a	0.434	<0.0001	9.64b	1.108	8.54b	0.623	5.90a	0.467	<0.0001
OFA	24.54	1.534	23.41	1.074	24.36	2.621	0.293	24.89	1.309	25.36	1.610	24.84	3.218	0.818
**FA Ratios**														
SFA/UFA	0.81	0.096	0.80	0.072	0.89	0.104	0.051	0.83	0.105	0.83	0.086	0.90	0.146	0.238
MUFA/SFA	0.65	0.110	0.71	0.098	0.73	0.102	0.177	0.64	0.116	0.71	0.122	0.71	0.149	0.358
PUFA/SFA	0.60b	0.040	0.56b	0.044	0.42a	0.049	<0.0001	0.58b	0.069	0.50ab	0.037	0.43a	0.042	<0.0001
PUFAn-6/PUFAn-3	1.47a	0.189	1.53a	0.158	1.86b	0.195	<0.0001	1.53a	0.151	1.45a	0.123	2.17b	0.250	<0.0001
**Nutraceutical Parameters**														
DFA	73.95	1.431	74.30	1.443	72.71	2.671	0.125	73.54	1.244	72.30	1.743	72.35	3.113	0.300
AI	0.47	0.063	0.41	0.049	0.45	0.092	0.097	0.46	0.065	0.48	0.067	0.46	0.105	0.770
TI	0.68a	0.081	0.68a	0.048	0.81b	0.095	<0.0001	0.70a	0.081	0.72a	0.068	0.85b	0.137	0.002
h/H	3.03	0.240	3.18	0.205	3.03	0.408	0.346	2.96	0.207	2.87	0.247	2.97	0.516	0.707
Δ9-desaturase C16	12.61a	2.468	13.79a	2.343	22.50b	5.765	<0.0001	11.32a	2.088	13.42a	1.726	21.10b	5.577	<0.0001
Δ9-desaturase C18	53.71	6.072	54.52	4.428	54.53	4.203	0.897	53.53	6.526	56.18	5.964	53.78	5.843	0.511
Elongase	62.96	2.565	62.93	2.128	61.42	4.492	0.417	62.27	1.840	61.67	3.213	60.71	5.035	0.572
NV	0.80	0.126	0.78	0.095	0.79	0.182	0.936	0.81	0.116	0.88	0.143	0.80	0.207	0.389
DHA+EPA	5.11c	0.118	3.65b	0.177	2.01a	0.160	<0.0001	5.34c	0.226	3.95b	0.090	2.27a	0.352	<0.0001
HPI	1.58a	0.169	1.81b	0.184	1.61a	0.254	0.018	1.64	0.221	1.55	0.238	1.62	0.331	0.682
CI	3.87a	0.158	4.08a	0.156	4.69b	0.370	<0.0001	4.03a	0.238	3.86a	0.065	4.81b	0.292	<0.0001
CSI	3.43a	0.165	3.59a	0.148	4.14b	0.392	<0.0001	3.56a	0.220	3.43a	0.080	4.27b	0.313	<0.0001

The different letters next to the mean value in the same row indicate significant differences between these values for each muscle. FFG—forest feeding ground; OFG—organic feeding ground; CFG—conventional feeding ground; Bold: Sums of FA Groups–sums of fatty acids groups; FA Ratios fatty acids ratios; Nutraceutical Parameters–nutraceutical indices of lipids.

**Table 5:** Contribution of fat, fatty acids, and cholesterol to an adult diet provided by a 100-g portion of red deer meat sampled from the three feeding grounds.

Specification	Energy Requirements (%) ^a^	g/Day (for a 2000 kcal Diet) ^b^	Mean Content (g/100 g of Fresh Meat)						Percent (%) of Contribution for a 2000 kcal Diet					
			*Longissimus Lumborum* (LL)			*Semimembranosus* (SM)			*Longissimus Lumborum* (LL)			*Semimembranosus* (SM)		
			FFG	OFG	CFG	FFG	OFG	CFG	FFG	OFG	CFG	FFG	OFG	CFG
Total fat	20.0–35.0	44.0–78.0	1.46	1.50	1.98	1.57	1.67	2.11	1.87–3.32	1.92–3.41	2.54–4.50	2.04–3.57	2.17–3.79	2.71–4.79
∑ SFA	<10.0	<22.0	0.471	0.478	0.673	0.512	0.544	0.771	≥2.14	≥2.17	≥3.03	≥2.33	≥2.47	≥3.26
∑ MUFA	15.0–20.0	33.0–44.0	0.308	0.337	0.478	0.326	0.385	0.543	0.69–0.95	0.77–1.03	1.11–1.47	0.74–0.99	0.87–1.17	1.13–1.54
∑ PUFA	6.0–11.0	13.0–24.0	0.281	0.267	0.276	0.294	0.274	0.325	1.16–2.17	1.12–2.06	1.15–2.12	1.23–2.26	1.14–2.11	1.26–2.34
∑ PUFA n-6	2.5–9.0	5.6–20.0	0.155	0.149	0.164	0.166	0.149	0.206	0.80–2.84	0.75–2.66	0.83–2.98	0.83–2.96	0.74–2.64	0.97–3.44
∑ PUFA n-3	0.5–2.0	1.1–4.4	0.107	0.100	0.089	0.109	0.103	0.096	2.43–9.72	2.25–9.00	2.03–8.09	2.47–9.91	2.34–9.36	2.03–8.09
EPA+DHA	250 mg	0.250	0.054	0.040	0.029	0.061	0.048	0.035	21.63	16.00	11.60	24.40	19.03	13.84
Cholesterol ^c^	<300 mg	<0.300	0.059	0.062	0.069	0.061	0.057	0.069	≥19.66	≥20.67	≥23.08	≥20.33	≥19.00	≥23.01

^a^ [49]; ^b^ [50]; ^c^ [51]; FFG—forest feeding ground; OFG—organic feeding ground; CFG—conventional feeding ground.

## Data Availability

The data presented in this study are available in the article.
